# A Polynomial Subset-Based Efficient Multi-Party Key Management System for Lightweight Device Networks

**DOI:** 10.3390/s17040670

**Published:** 2017-03-24

**Authors:** Zahid Mahmood, Huansheng Ning, AtaUllah Ghafoor

**Affiliations:** 1 School of Computer and Communication Engineering, University of Science and Technology Beijing, Beijing 100083, China; b20140561@xs.ustb.edu.cn; 2 Department of Computer Science, National University of Modern Languages, Islamabad 44000, Pakistan; aullah@numl.edu.pk

**Keywords:** path key, symmetric, polynomial, hashing, proxy node, node storage

## Abstract

Wireless Sensor Networks (WSNs) consist of lightweight devices to measure sensitive data that are highly vulnerable to security attacks due to their constrained resources. In a similar manner, the internet-based lightweight devices used in the Internet of Things (IoT) are facing severe security and privacy issues because of the direct accessibility of devices due to their connection to the internet. Complex and resource-intensive security schemes are infeasible and reduce the network lifetime. In this regard, we have explored the polynomial distribution-based key establishment schemes and identified an issue that the resultant polynomial value is either storage intensive or infeasible when large values are multiplied. It becomes more costly when these polynomials are regenerated dynamically after each node join or leave operation and whenever key is refreshed. To reduce the computation, we have proposed an Efficient Key Management (EKM) scheme for multiparty communication-based scenarios. The proposed session key management protocol is established by applying a symmetric polynomial for group members, and the group head acts as a responsible node. The polynomial generation method uses security credentials and secure hash function. Symmetric cryptographic parameters are efficient in computation, communication, and the storage required. The security justification of the proposed scheme has been completed by using Rubin logic, which guarantees that the protocol attains mutual validation and session key agreement property strongly among the participating entities. Simulation scenarios are performed using NS 2.35 to validate the results for storage, communication, latency, energy, and polynomial calculation costs during authentication, session key generation, node migration, secure joining, and leaving phases. EKM is efficient regarding storage, computation, and communication overhead and can protect WSN-based IoT infrastructure.

## 1. Introduction

A recent advancement in communication technology, Wireless Sensor Networks (WSNs) are widely used in several applications [1]. The science community has focused on the security of WSNs. Because of the resource-constrained environment of the WSNs, classic security mechanisms are not practical since they consume too much energy; hence researchers are proposing new lightweight security mechanisms for every possible security aspect of WSNs. WSNs consist of many small, low-cost, self-governing ends called sensor nodes with little ability to manipulate data [2] and with constrained computing, energy, and memory. MICA2 Motes use 8 bit, 16 MHz processors with 4 K bytes of electronically erasable programmable read only memory (EEPROM) and 128 K bytes of programmable memory [3]. With the passage of time, the sizes of WSN are growing in clusters [4]. Traditional security mechanisms using public key cryptography cause significant overhead regarding computation and communication. Key management is mandatory and more challenging with limited resources in WSNs [5]. Many researchers have been recognized that grouped or dispersed heterogeneous sensor systems can sensibly perform with system effectiveness, operational execution, and enduring system lifetimes.

**Figure 1:**
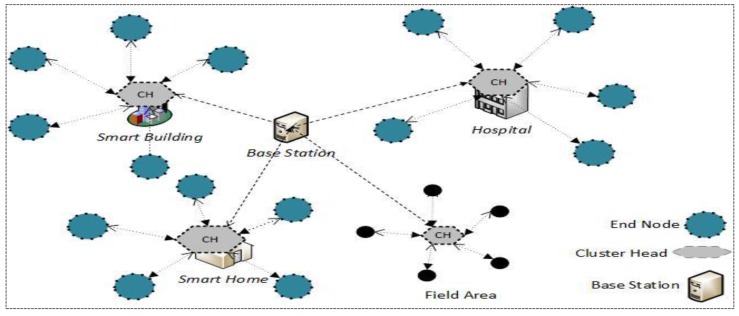
Heterogeneous WSN-based IoT architecture.

In a heterogeneously grouped approach, as described in Figure 1, the low end sensor (*L*-sensors) are resource-constrained devices with low power, short communication range, limited memory, and less computation power. On the other hand, *H*-sensors are equipped with tamper resistance and have enough resources, like high battery power, broad communication ranges, sufficient memory, and high computational capabilities. *L*-sensors are deliberately conveyed in a group, and every group is controlled by a group head (*H*-sensor). The *L*-sensors essentially sense environmental statistics and forward it to the *H*-sensors and the other way around. *H*-sensors can perform complex operations on the sensor information, and utilize longer radio and straightforwardly transfer it to the base-station. The base-station (BS) is a powerful hub, and it has abundant sources. The BS might be a remote server, and it might be connected with the external world utilizing the accelerated Internet.

Various anonymous security protocols have been recommended for heterogeneous WSNs in recent past, and they display diverse sorts as well as levels of security protection at various cost. In this section, we discuss the existing cryptography techniques that addressed the issue of authentication in heterogeneous WSNs. Due to the limited resources of WSNs, a sound balance between security level and the associated energy utilization overhead is needed to mitigate the security threats. Symmetric parameters such as node ID, message authentication code, nonce number, and time stamps are the significant parameters for grouped key management techniques which are energy efficient and avoid the different type of attacks from malicious nodes and avoid node compromising attacks. Most security protocols developed for WSNs use symmetric encryption, due to ease of its implementation [6]. Besides this, single node authentication has become unable to meet the increasing communication demand, as the service demand increases with the passage time, multiparty computing is necessary for node authentication simultaneously and securely inter-cluster and intra-cluster where [7] participants collectively to set up a mutual session key to enable the multi-party computing and secure exchange of messages.

Multi-party computing for security credential calculations has been adopted using many-to-many nodes communication scenarios. Conventional node authentication involves computation at single sensor node for the entire security credential calculation, which is less secure as compared to trustworthy multi-party involvement. These days, a new form of authentication, known as group authentication [8] is proposed which may be used to decide whether or not all users belong to the same group or now not. The group authentication is very green for the reason that it can authenticate all members at one time. It can be used for pre-processing of node authentication to identify trustworthy contributors and non-contributors as well.

To communicate securely with the same group and among different groups, we employed a polynomial (P) to achieve efficient intra-group key refreshment and generated a polynomial to create an inter-group key. For this reason, our proposed protocol could be highly effective in each authentication and key establishment because it may provide the most effective broadcast transmission. Moreover, for computations every sensor needs a polynomial assessment and key-hash feature and it is easier for performing encryption and decryption. In [9] a technique to optimize the energy consumption for computing polynomial expressions was proposed. In this context, it is, therefore, crucial to have efficient key revocation and renewal mechanisms in WSNs. The goal of this work is to propose secure and efficient protocols for key revocation and renewal.

This research proposes an Efficient Keying for Multi-party (EKM) scheme to calculate the polynomial values using lightweight *XOR* operations. It also includes pre-deployment, node joining, and key establishment protocols. Before the nodes’ deployment in WSNs, each sensor node is pre-configured with a set of symmetric keys shared with all the other sensor nodes of the network to transmit IDs securely. After the network deployment, every sensor node identifies the specified symmetric keys used to communicate with the cluster head (*CH*). The cluster-head maintains all the symmetric keys shared with the sensor nodes belonging to its cluster. The main reason the use of these symmetric keys is to facilitate the multi-hop communication while transmitting secret data, particularly, the personal-proportion distribution and the exchanged facts among the cluster-heads and the base station (BS). The implementation of the polynomial that is applied for deriving an intra-group key can reduce the session key storage overhead at the member nodes and their *CH*. After the intra-group key is acquired, the member nodes self-generate the polynomial functions, which are necessary for growing an inter-organization key. This facilitates the reduction of the communication overhead on the *CH*. We have proposed a key generation and update mechanism for secure data sharing in identical clusters and among different cluster. In the session key generation mechanism for intra-clusters, the proposed technique significantly decreases the number of broadcast messages in the inter-cluster communication. The extra number of re-keying messages and communication overheads due to the proposed scheme has been conscientiously justified. Through the usage of the proposed mechanism, we have enhanced the WSN efficiency and network lifetime by reducing the number of exchanged messages to enhance the network lifetime during node mobility while reducing the coverage area of cluster heads and the need to migrate nodes to join the powerful coverage area of the *CH*. This research proposes a secure node migration protocol in which reliable handoff occurs and new connections are established between *CHs* and member nodes.

The organization of the rest of the paper is as follows: the system and security model for proposed protocol established in Section 2. Section 3 elaborates the related work to develop existing key establishment schemes. Section 4 provides a detailed discussion on a proposed EKM system for key distribution, node joining, and key establishment scenarios. The formal specification and security analysis of proposed technique is discussed in Section 5. Section 6 are about simulation parameters and scenarios to present results and illustrate the analytical discussion. Finally, Section 7 provides conclusions and future work.

## 2. System and Security Model

**Figure 2:**
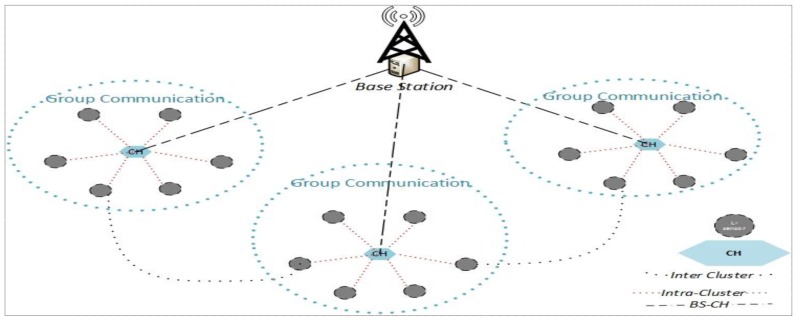
Network model of multi-party key establishment scenario.

In this section, the network model, adversarial model, and security model are described, respectively. For multicast communications in WSNs, the network model used here is the heterogeneous and hierarchical topology depicted in Figure 2. We assume that the network consists of a sink and many groups, and each group has a powerful node which has abundant resources (energy, computing, and storage) and many low ordinary nodes. The powerful node acts as the cluster head (*CH*) and is responsible for network security management, such as key issuing, updating, revocation, intrusion detection, etc. As an ordinary member, *L*-sensor is responsible for sensing data and authentication for secure communications.

When we consider that WSN may additionally encounter attacks from inside by compromised nodes or external attackers, so its lifetime can be classified into many phases. At the beginning of every session, all types of keys like BS-*CH*, *CH*-*L*-Sensor, and *L*-Sensor-*L*-Sensor for security functions will be re-issued. The length of every session could be modified dynamically with the security strength of the safety mechanism used or the regulations of group members like new node addition and migration. Session switching might be launched using cluster heads for group members and by the BS for cluster heads. The self-restoration key control schemes proposed in particular focus on the problems, updating and revocation manner of organization keys and pairwise session keys. Different styles of keys methods may be originated according to the devices for group keys and pairwise keys.

### 2.1. Threat Model

In this section, we describe the threat model adopted in the proposed scenario. We assume that there aren’t any compromised nodes at network deployment time. Attacks are released a while after the nodes are deployed. With effective computing capability, the adversary can execute advanced fact evaluation to gather the important data. The adversary can be either inner or external and active or passive. Moreover, a single entity or a member of a coordinated group of entities can also perform malicious activities. An adversary can launch a physical attack by compromising a node and reading secret data to use it for future tracking. An insider adversary is a malicious node that may examine/alter all messages encrypted with its keys. Passive adversaries can spy on all communication channels in the community like idea channels, or unintended verbal exchange channels. Energetic adversaries can examine, tamper, and inject fraudulent packets over verbal exchange channels.

#### 2.1.1. Adversarial Model

Moreover, insider attackers and outside attackers can apportion the important thing, captured information, to interrupt the security collusively, in what is referred to as a collusive physical attack. For instance, the revoked nodes and the newly joined nodes can launch deceitful attacks on the session keys no longer belonging to them. We expect that the compromised or insider adversary behaving abnormally could be revoked from the network inside the following session with the assist of the intrusion detection mechanism, which is not addressed in this research.

Moreover, a comprehensive attacker can easily monitor the wireless data of WSNs and capture the wireless packets. The attacker will be able to gather plenty of useful sensor information from the captured packets like node IDs and node location and might adjust packet information even as a packet is in transit. Additionally important, an attacker can intentionally apprehend a node and capture all its cryptographic keys. As a result, node capturing physical attacks are very harmful to the network if excessive numbers of cryptographic keys remain stored on a node.

#### 2.1.2. Potential Attacks

In general, attacks affecting secure key establishment are eavesdropping and node capture [10], referred to as passive adversaries and active adversaries. Passive adversaries gather some statistics via eavesdropping without any physical access to a network entry, and active adversaries encrypt information that is leaked during physical access to the node. The proposed key management technique is secure against eavesdropping and node capture, and we will explain the cause for this below. During cluster key derivation, the cluster head sends an expanded polynomial without any encryption. It is not easy to guess the intra-group key management from the polynomial, considering that it is hard to do polynomial factorization due to the fact there may be certainly a $O(n\left((log_{n}\right)$) options to the polynomial enlargement problem [11,12] and the problem for polynomial factorization is NP-hard [13]. For example, $P=\left(3x^{3}+\;15x^{2}+74x-117\right)$ is not easy to factorize as P=〈x−5)|(x−4)|(x−6)+3〉 for the purpose, it’s tough to guess 3. Even though the accelerated polynomial P is sent without any encryption, cluster head key credentials will no longer leak out. For this reason, it’s far tougher to generate the coefficient of polynomial H(x) with the aid of an attacker due to the safety of these keys in clusters.

### 2.2. System Model

Based on the system and attack model, we particularly grasp the subsequent security goals: for the reason that *L*-sensors aren’t truthful, initializing essential keys allows powerful security. To preserve the mutual authentication among the network devices (sensors, cluster-heads, and base-stations), devices have to perform mutual authentication and establish a dynamic session key before establishing the consultation, so that each the nodes/gadgets can confirm their legitimacy. In vital applications (place of birth security, healthcare, smart grids), nodes’ identities are very critical, as a consequence, nodes’ identities should be stored securely and privately [14,15,16]. To protect the Wi-Fi communication from unlawful eavesdropping and interception all the wireless messages should be secured (i.e., personal), so that a global adversary can’t examine, reveal, intercept, or modify the Wi-Fi messages. During an adversary monitoring, the *L*-sensors are easily compromised, so then a key revocation mechanism can play a vital function to protect from any future misleading moves from a compromised node. Moreover, a node addition feature confers scalability to the network.

The proposed model for our key distribution scheme is designed for heterogeneous sensor networks. We assume a heterogeneous sensor network consists of high resource node (*H*-sensors) and their member nodes having fewer resources called *L*-sensors. *H*-sensors are nodes that are more powerful and have more computation, communication, energy supply and storage capability’s sensors are ordinary sensor nodes with limited computation, communication, and energy supply and storage capability. We consider the hierarchical structure of the heterogeneous sensor network proposed in [17] in which high power sensor nodes work as cluster heads and low power sensor nodes work as their member nodes. This sensor clustering scheme enables local data processing, which reduces the communication load in the network to provide scalable solutions. To increase the lifetime of the deployed network the nodes are divided according to their workload. Initially, when a sensor network is deployed, there are large number of low power sensor nodes and a limited quantity of cluster heads. It is assumed that the *H*-sensor and *L*-sensors are deployed uniformly and randomly. It has been assumed that the *CH* is tamper resistant and cannot be compromised. The major issue with the polynomial calculation is that resulting multiplicative values become very large and slow for larger values of polynomial factors. Due to constrained resources, when the number of *L*-sensors increases then the computation and communication cost to the cluster head also increases. The more *L*-sensors there are, the more memory required on the cluster head for restoring their IDs and multiplicative values, which causes memory overhead.

## 3. Related Work

Different issues with existing key management techniques are discussed in conjunction with the availability of limited recourses of WSNs. Minimizing the resource consumption and maximizing security levels are two different points. The probabilistic scheme described in [18], where each node receives a group of keys smaller than the network size provides better connectivity and little memory overhead. It has key pre-distribution, path-key establishment, and shared key discovery phases that cause scalability problems. The larger network size for a given stage increases the computation and communication overheads. The secret key sharing is limited by the memory existing on the sensor nodes, since the chance that next node shares k/n give a *k*ey, for example, the memory overhead is *O*(*n*)*.* For fixed key chain size, the key connectivity achieved is lower than previously.

**Figure 3:**
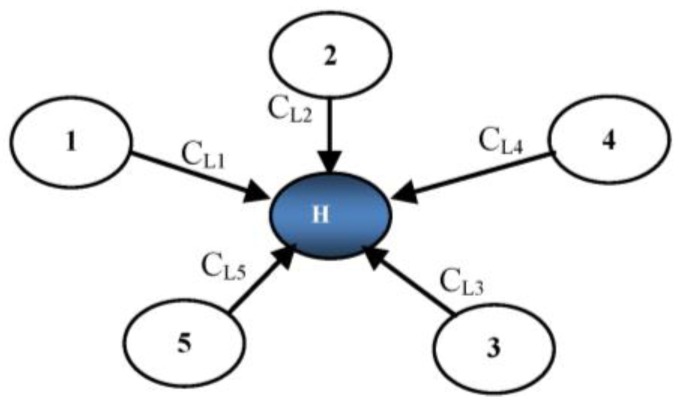
Polynomial computation at a *H*-sensor.

In [19] a dynamic generation of the polynomial (DGP) is proposed for heterogeneous WSNs scheme where nodes are pre-loaded with a master key *K* for secure communication during cluster key establishment. During the key management process, each node calculates *MAC*(*ID*) and then encrypts the concatenated message with the master key. Each *L*-sensor node (*L_i_*) generates ciphertext *C__Li_ = E_MK_*(*ID||MAC*(*ID*)) and transmits it towards a *H*-sensor as depicted in Figure 3. The *H*-sensor calculates the hash of *L*-sensor *IDs* and saves these values. *H*-Sensor generates a polynomial using Equation (1) where *h*(*Li*) is hashed function for *L*-sensor *Li* and *i* = 1, …, *n.*

(1) $$(x-\;h\left(l_{1}\right)\left(x-h\left(l_{2}\right)\right).\;.\;.\;\left(x-h\left(l_{n}\right)\right)K_{\mathrm{chj}}$$

During a node joins or leave operation, *H*-Sensor revokes existing polynomial and broadcast the new polynomial to all *L*-Sensors by taking *MAC*(*ID*) of all *L*-sensors. It increases the lifetime of sensor networks by decreasing the key pool size. Our scheme further reduces the computational cost of this scheme.

In [20] where each node receives a unique ID, a private key, and a master key *K*. After deployment, sensor nodes broadcast their IDs encrypted with the global key. A loop is constructed by using a certain number of a node from set *S*. If sensor node *A* creates loop *L* with set *S*, then loop-key KL=Hash(ts||KA||IDA||{ID}) is computed. The global key protects the distribution of the loop key inside the loop. A network-wide key can reduce communication, but capturing a single node exposes all the links in the network. Our approach prevents it from taking the credentials from member nodes.

In [21] every sensor node randomly picks up a unique secret key from the large key pool and produces a new secret key by applying one-way hash function. The *H*-sensor broadcasts a message in its communication range and then, the *L*-sensors discover their neighbors. It provides complete connectivity, resilience against sensor node capture and routing attack. Our scheme follows the same deployment pattern. Sajid recommended following a sequence of Key Pool → Key Chain→ Key Ring [22]. It achieves an equal probability of key sharing among nodes and stores a small number of key generations on the node. It reduces the memory overhead if the wrong parameter is fixed, but for maximum key sharing, it needs a larger pool size.

In [23] a secure symmetric key used for member nodes and public key cryptography for powerful nodes was proposed. Three scenario-based schemes reduce the memory and computational overhead along with high resilience against node capture attacks. A key management regime described in [24] used a keyed hash chain. It supports the establishment and renewal of five keys, and it also overcomes the drawback of a single key. A critical decision is to how determine the length of a key-chain. A large key-chain size increases the memory overhead and on the other hand, decreasing the chain size compromises the security strength.

A random key pre-distribution scheme where seeds are taken from the base station to drive a new key that is providing adequate security was described in [17]. It allows privately distributing a secret to a set of recipients with only one multicast communication [25]. A lightweight *XOR*-based re-keying scheme is illustrated in [26] that does not require message exchange from common nodes. In this system, *CH* transmits a Magic code and a nonce number in the encrypted message to all member nodes. Sensors take *XOR* of an existing key with a left shifted Magic word and get a new value that is further used to establish *XOR* with the nonce. Similarly, our scheme also supports key freshness on each node join or leave operation. A technique put forth in [27] enforces mutual authentication between a member and group key distributor while executing a re-keying process.

During the last decades, various applications have been benefitting from WSNs, such as environmental monitoring, battlefield intelligence, and reconnaissance, medical caring, intelligent home, industrial control [28]. However, in some harsh and critical environments, malicious attacks, including eavesdropping, Denial of Service (DOS) attacks, and packet tampering and infection, may disturb the network operations and degrade the system performance. Security issues should be considered carefully during the design of WSNs’ protocols to defend against and tolerate attacks [29]. Cryptography is typically used to provide security features, such as confidentiality, authentication, integrity, and availability [30]. As the core role of security, key management is responsible for key distribution, updating, and revocation for sensor nodes, and establishes security associations between nodes [31]. As per scientific techniques, some related works explore polynomial [32,33] vector space secret sharing [34], bilinear pairings [35] and elliptic curve cryptography [36]. Polynomials can be used to improve the trade-off between security performances, computation complexity, and resource consumption better than other methods [37]. In this paper we concentrate on a polynomial-based strategy to achieve better system performance.

References [38,39] elaborate that radio-frequency identification (RFID) is a program distinguishing proven innovation and a promising innovation to be naturally incorporated into conventional remote sensor systems (WSNs) including a novel recognizable proof measurement into WSNs and forming RFID-based sensor frameworks. The lightweight protocol provides the security properties because basic logarithmic and intelligent operations without requiring costly cryptographic calculations are implemented in low-cost and resource-restricted wireless systems.

The scheme in [40] enforces mutual authentication between a member and the group key distributor while executing the rekeying process. It provides forward secrecy and backward secrecy properties, and resists replay attacks, impersonation attacks, group key disclosure attacks and malicious insider attacks. The proposed scheme can be used for rekeying purposes in a secure multicast scenario. The second one is an authentication mechanism to be used in environments in which a public-key infrastructure is not available. Forceful characteristics such as changing the key of a user in a chain of command influences the performance of an admittance organize scheme more than other dynamic properties such as adding or deleting a security class to the hierarchy [41]. To overcome forgery attacks, the authors focuses on the security of the data provenance specific to a wireless sensor network. The paper provides a scheme to detect forgeries in provenance data, detection of packet drop attacks and also identifies the attacker of packet drop attacks [42]. This scheme used a packet bloom filter data structure for encoding provenance data. Hash functions are used to encode the provenance data in packet bloom filter. The use of a bloom filter data structure results in a lightweight scheme which is suitable for wireless sensor networks. Each packet consists of the sequence id of the packet, actual sensor data, and an n-bit bloom filter vector. To detect a forgery attack, the ID of the each node in the path is encoded into the bloom filter vector. It is assumed that base station knows the path of the received packet. When a packet is reached at the base station in the WSN, a fresh bloom filter is taken and encoded with all nodes in the path. If the generated bloom filter and bloom filter extracted from the received packet are the same, then there is no forgery attack, and otherwise there is a forgery attack. Actual sensor data is also secured along with provenance data with the scheme proposed in this paper. Experimental results proved the effectiveness and the lightweight nature of the scheme.

## 4. Efficient Keying for Multi-Party Scheme (EKM)

In this phase, mutual authentication and session key establishment takes place between the participants. During cluster key establishment, an *H*-sensor or cluster head (*CH*) receives encrypted IDs of *L*-sensors from neighboring nodes. *CH* decrypts these IDs using the pre-distributed master key K. After receiving the IDs, a hash of these IDs is calculated and stored in memory as illustrated in Figure 3. *CH* selects random IDs from the stored hash list to compute the polynomial. Finally, the polynomial is concatenated with its MAC and encrypted using a master key to transmit towards all neighboring nodes. In our scheme, we considered the hierarchical structure of [20] for the deployment. It has been assumed that the *H*-sensor and *L*-sensors are deployed uniformly. It is also presumed that the *CH* is tamper resistant and cannot be compromised. A list of the notations for EKM is provided in Table 1.

**Figure 4:**
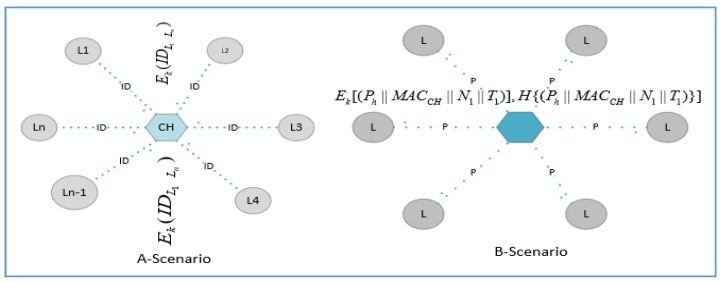
Intra-cluster key establishment.

Every common node has been preloaded with a hash function, master key, encryption and decryption functions along with a unique ID by the sink. The *CH* is also pre-loaded with similar credentials along with a polynomial calculation function. The *CH* and sink server have secure authentication mechanisms, including *ECC* which is hard to reverse. The *CH* forwards this request after making it secure to s sink that verifies the request and responds to the *CH* granting or denying this request. Figure 4 shows that the *CH* receives an encrypted message *E_KM_*(*ID_Li_||MAC*(*ID_Li_*)) containing the ID of *Li* and *MAC*(*ID_Li_*). The *CH* selects the random IDs and hashing to calculate a polynomial using Equation (3). Finally, the *CH* transmits the encrypted message *E_KM_*(*Ph||MAC* (*Ph*)) to the *L*-sensors.

Initially, the polynomial $P_{h}$ is calculated by using Equation (2) where x is a pre-defined significant value, and the hashes *h* are calculated for all member node identities from $ID_{L_{a}}$ to $ID_{L_{a+\gamma }}$ where γ be the cluster size. We further reduced the computation overhead by taking *XOR* of a randomly selected number of hash values instead of taking the *XOR* of all values. The *CH* generates the hashes of all IDs received from neighboring *L*-sensors using the initialize procedure.

After the hashing calculation, the *CH* makes a polynomial *P_h_* by taking *XOR* of randomly chosen *R* hash values as shown in Equation (3), where *a*, *b* and *c* are indices of randomly selected node IDs for *R* = *3*:

(2) $$P_{h}=(x-h_{a}\left(ID_{L_{a}}\right)\;XOR\;\left(x-h_{a+1}\left(ID_{L_{a+1}}\right)\right)...XOR\left(x-h_{a+\gamma }\left(ID_{a+\gamma }\right)\right)$$

(3) $$P_{h}=\;(x-\;h_{a}\left(ID_{L_{a}}\right)\;XOR\;\left(x-\;h_{b}\left(ID_{L_{b}}\right)\right)XOR\;\left(x-\;h_{c}\left(ID_{c}\right)\right)$$

**Figure 5:**
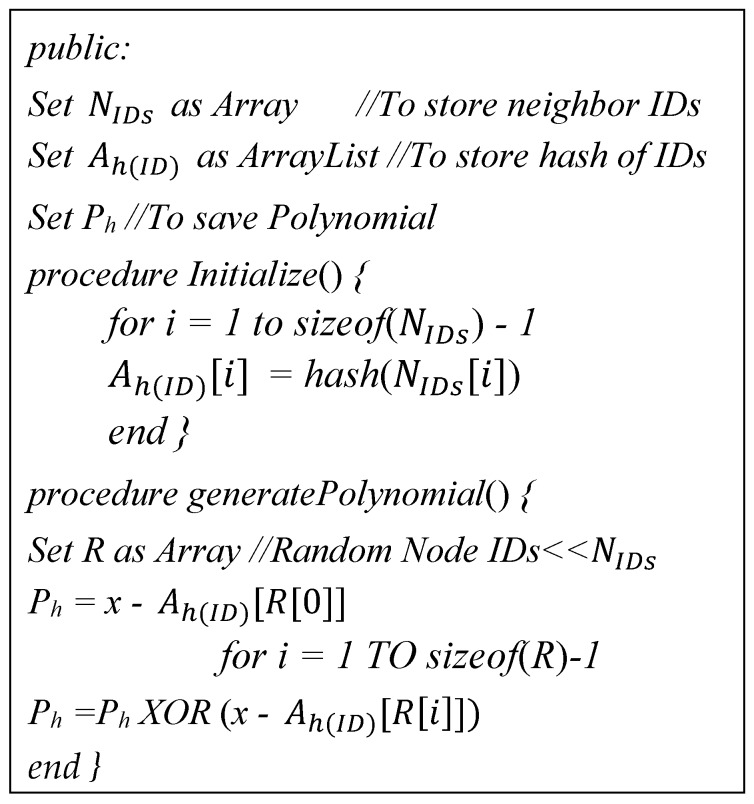
Polynomial calculation algorithm.

Moreover, the algorithmic steps of Equation (3) are illustrated in Figure 5. We recommend using *R = 3*, i.e., randomly taking three hash values and using *XOR* to generate the polynomial. It is enough to create confusion for the attacker in the selection of *IDs* out of *N_IDs_*.

### 4.1. Addition of New Node

**Figure 6:**
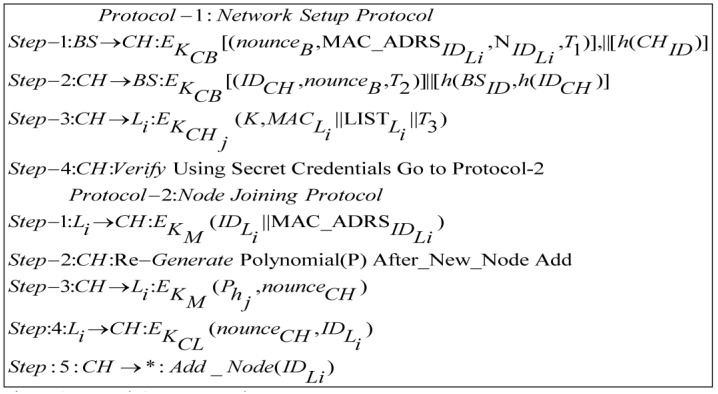
Network setup protocol.

**Figure 7:**
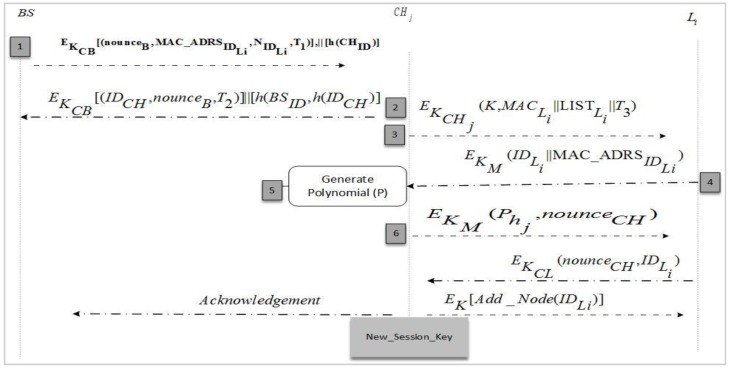
Node addition protocol flow diagram.

After deployment of WSN, the addition of a new sensor node in the cluster may also occur. For new node addition, the base station plays a vital role in the authentication process and performs the setup phase. Into the network setup model phase, the *BS* introduces the new sensor to the *CH*. The sensor node is pre-loaded with the master key (*K*), a one-way hash function *h*(*x*) and a randomly selected node id ${\mathrm{ID}}_{L_{i}}$. After that, Bthe S broadcasts an encrypted message containing$\;{\mathrm{nonce}}_{B}$, $N_{{\mathrm{ID}}_{\mathrm{Li}}}$ (list of *L*-sensor IDs) and $\mathrm{MAC}\_{\mathrm{ADRS}}_{{\mathrm{ID}}_{\mathrm{Li}}}$ to all cluster head nodes as illustrated in Protocol-1. *CH* replies with an encrypted message containing ${\mathrm{ID}}_{\mathrm{CH}}$ and${\;\mathrm{nounce}}_{B}$. The cluster head multicasts the session key, list of member nodes and MAC in encrypted form. This ensures the originality and reliability of the node joining process. The new node addition protocol and flow diagram are depicted in Figure 6 and Figure 7, respectively, where before new node addition *CH* get an authentication from the BS. After successful authentication, *CH* runs Protocol-2 for verification using secret credentials.

The new *L*-sensor is deployed randomly in the sensor network area. The new *L*-sensor encrypts its id and $\mathrm{MAC}\_{\mathrm{ADRS}}_{ID_{Li}}$ through its pre-loaded master key *K_M_*. The cluster head, which already has a list of new sensors, verifies it by comparing the received *MAC* with its list. After verification, it replies with polynomials $P_{h_{j}}$ encrypted using *K_M_*. The new *L*-senor decrypts the message and gets a polynomial to calculate a cluster key $E_{K_{CL}}$ between *CH* and $L_{i}$. Finally, *CH* informs all members to add $ID_{Li}$ in member list as illustrated in Protocol 2. If a new node is a malicious node, then the *CH* sends an adversary identification message to the base station. After the deployment in a heterogeneous network, new nodes are placed in such a position where they can communicate directly with the cluster head. Figure 6 shows the overall scenarios of Protocol-1 in steps 1 to 3 whereas Protocol-2 is in steps 4 to 7.

### 4.2. Migration of Node

**Figure 8:**
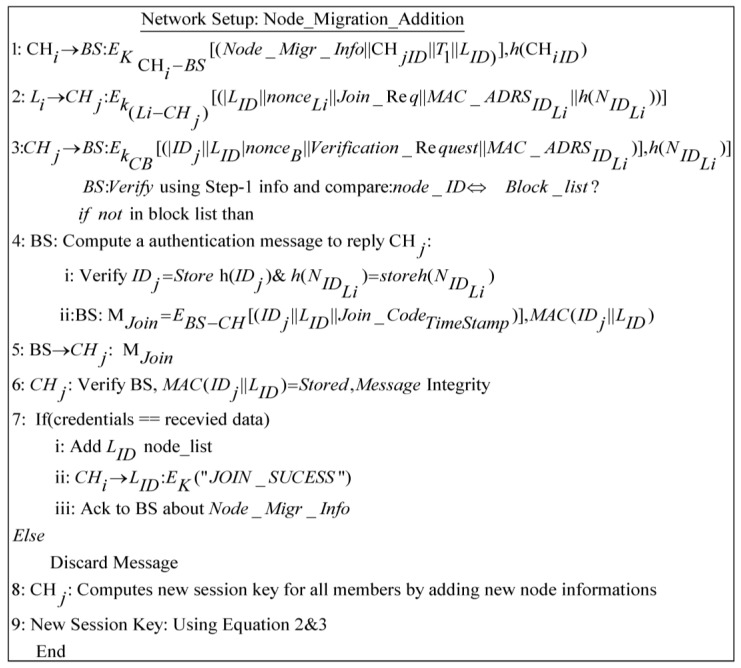
Node migrations and addition protocol.

**Figure 9:**
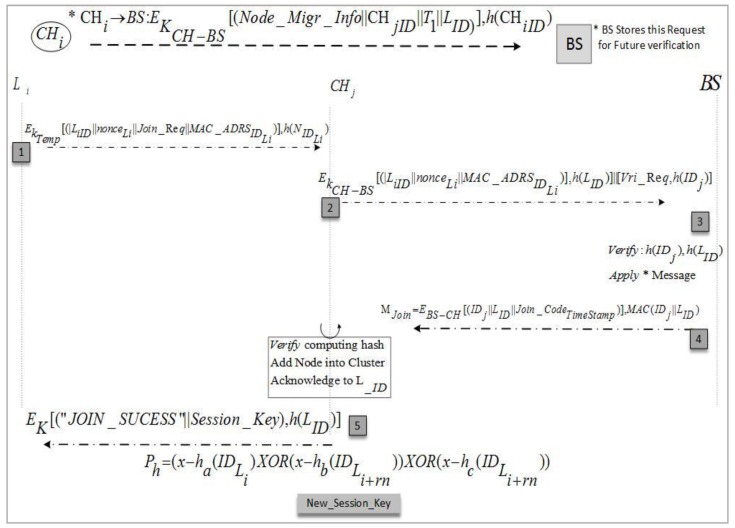
Node migration setup and new intra-group session keys.

To increase the lifetime of a WSN network, we have proposed a secure node join protocol for moved nodes. The main purpose of this phase is to add displaced nodes to existing clusters by the *CH* sensing range in their locality. There are instances in which a node can belong to two clusters, inducing a loss of capability in the different one, which will resolve this issue; the cluster heads that do not have a specific sensor kind in their clusters invite the missing sensor to join or suggest another *CH* according to its sensing range. This achieves better security in an environment where nodes are mobile and leave/join the cluster frequently. We have maintained a binary search tree for all network nodes at the BS that is aware of each leave and join operation. Similarly, a Binary Search Tree (BST) is also maintained at the *CH* to keep the node list updated and maintain a record of leaving nodes. It also keeps a list of rejected nodes which do not have security credentials as per issued by BS. This guards against intruders joining the network during migration from other networks. We have supposed that an intrusion detection system is working to identify those nodes that were trustworthy but now are performing malicious activities due to some attack. Such nodes are also added to the rejected list. Due to this record keeping, the *CH* intimates in a timely way the nodes that want to send a message to a node that has left the network or been set as malicious to saves extra communication costs at low-powered nodes. During the node migration and addition scenario, the protocol in Figure 8 is described in a stepwise manner in following section. Figure 9 elucidates these protocol steps in a node wise interaction scenario.

Steps 1–3Before node migration, *CH_i_* sends a node migration notation to BS with the valid ID of a sensor node that needs to migrate. A migrated node sends a joining request to join new cluster *CH_j_* with a pre-shared key and transmits secret credentials for the joining process. Upon receiving a migrated node joining request, the *CH_j_* get verification from the BS by sending a verification request. The BS verifies or rejects the node joining procedure by comparing said node’s identity in the block node database.Steps 4–5After the migrated node joining request verification, the BS computes an authentication message for *CH_j_* and transmits it in encrypted form using a shared key.Steps 6–7The *CH_j_* verifies the message credibility using secret parameters before adding the migrated node in its cluster. Carrying out verification, *CH_j_* sends a join success message to the migrated node and an acknowledgment bit to the BSSteps 8–9Upon the migrated node successfully joining, *CH_j_* computes a fresh polynomial using Equations (2) and (3), and updates the cluster key for future communication and process aborts.

## 5. EKM Formal Specification

We have carried out aspect modeling the use of Non-monotonic Cryptographic Protocol (NCP), as well referred to as Rubin logic [43] by analyzing and verify the EKM protocol as much as possible through formal specifications. It validates the protocol as approximating through standard requirements about cryptographic operations like node authentication, message integrity, message freshness, encryption, decryption, etc. It additionally helps to become aware of any deficiencies within the proposed protocol and possibilities regarding compromise attacks. It is close to the real implementation and flow of programming functions. In Rubin logic, the entities are allocated roles and a global set is additionally maintained where data about the protocol is maintained in sets then these also keep updated states on the users after every updateable operation. Global sets are available in conformity with entire the part nodes, however, may stand labeled as secret, observer, rule and essential sets.

A local set is maintained at each entity or node and can be classified into possession POSS(), belief BEL(), seen and behavior sets BL(), the explicit specifications from [13] of these sets investigated. By applying Rubin logic on EKM, the local set involved is in Table 2, and then its verification and analysis part provides a detailed overview of all sets continue under the grouping of the local set. Local sets for all the participants including sender, receiver and actor nodes are independently managed. A possession set POSS (entity) holds all the constraints involved in message encryption, decryption and other procedures executed at a local RAM of each entity as described in the section below. A behavior list BL() holds the list of operations and input arguments that are performed in near to execution steps by entities *L_i_*, *CH* and BS.

The detailed analysis of the proposed scheme is presented in Table 2 by considering all communication actors. The sensor node $L_{i}$ authenticates itself to its cluster head by sending a join request message containing $Hash\left(h\left(.\right);\;ID_{Li}\right)$. Upon receiving this message, $CH_{i}$ computes polynomial $P_{Li}$ and reply$\;C_{1}$ in encrypted message $M_{1}$ to $L_{i}$. Upon receiving $L_{i}$ splits the received message and verifies it by getting *CH* and its information for joining a group. On the other hand, $CH_{i}$ contains its own (ID), member node (IDs), master key, and encryption key to communicate with$\;CH_{i}$ and member node *L_i_* in its possession. In$\;CH_{i}$, we believe the received messages and participant information are reliable and trustworthy. Upon receiving credentials from member nodes, $CH_{i}$ decrypts them by using the corresponding secret key and verifying the information after decrypting the ciphered text. Based on strong verification $CH_{i}$ accepts or aborts the member node join, leave and migration requests as well as communicates with the BS. The third and the most important entity of this scenario is (BS), which has more computation, communication, and storage power. $CH_{i}\;$possess their own (ID), CH(IDs), secret keys and a list of member nodes with their (IDs) It believes it has secure communication mechanisms for $\left(\mathrm{BS}\right)-CH_{i}$, $\left(CH_{i}\right)-CH_{i}$ and strong, secure key generation protocols. It verifies, $CH_{i}$ sensor nodes, and their authentication credentials and the validities of their identities.

### EKM Analysis and Verification

In this subsection, EKM is broken down for a node migration situation as discussed in Section 4.2. In this case, the key circulation is started by the base station that preloads the security credentials in *L_i_* and then (*L_i_*) transmits a message $M_{1}$ to the cluster heads $CH_{j}$. After receiving the message, *CH_j_* gets the node ids of participant sensor node and calculates the shared session key. Upon the completion of the transmission operation, an update operation is performed to refresh the spectator list demonstrated as follows. Related parameters including messages, ciphers, nonce values, actual key, and hashes are saved in possession set at *L_i_* during the key establishment phase as shown below. At the end of the migration, the forget operation removes the temporary values used in operations including $nonce_{Li}$, $P_{Li},\;H_{Li},\;C_{1},\;M_{1},M_{4}.$

*POSS*(*L_i_*) = *{ID_Li_, K_Li − CHj_, nonce**_Li_**, P_Li_, H_Li_, C_1_, M_1_, M_4_}*

After receiving messages, *CH_j_*, decrypts them to retrieve a concatenated message that is further split to get the actual parameters. The possession set at *CH_j_* contains the following parameters during execution of the abovementioned operations. The forget operation will result in removing the temporary values including $P_{CHj}$, nonceLi*nounce*, $C_{1}$, $C_{2}$C, $M_{2}$M, $C_{3}$C, $M_{4}$, $H_{CHj},P_{CHj\sim },nonce_{CHj}$ nounce from the possession set:

*POSS*(*CH_j_*) = {*ID_CHj_*, *K_CHj−Li_*, *K_CHj−BS_*, *P_CHj_*, *nounce**_Li_***, *C*_1_, *C*_2_, *M*_2_, *C*_3_, *M*_4_, *H_CHj_*, *P_CHj_*, *nounce_CHj_*, *ID_BS_*, *ID_Li_*}

The BS receives the message from *CH_j_* and after decryption, it verifies the *L_i_* and its security credentials. The possession set values at the BS contain temporary values as shown below. Forget operations are performed to remove the values *P_BS_*, *C*_2_, *C*_3_ and *M*_3_:

*POSS*(*BS*) = {*ID_BS_*, *K_BS−CHj_*, *P_BS_*, *C*_2_, *C*_3_, *M*_3_, *ID_Li_*, *Join-Code_T_*}

The receiver node BS verifies the credentials sent by the *CH_j_* and then returns a message containing *Join-Code_T_* to *CH_j_*. Finally, the possession set at *L_i_* is refreshed after the completion of node migration from *CH_i_* and successful joining at the *CH_j_*.

## 6. Results and Analysis

The proposed scheme is simulated using NS2.35 using C on Fedora core-12. TCL is utilized for simulating the nodes in multiple clusters. *H*-sensors are separately configured for an initial energy of 10,000 Joules, and message transmission and receiving has a cost of 0.5809 J and 0.049 J, respectively. The transmission radii of the *H*-sensor and *L*-sensor are set at 300 m and 50 m, respectively. We deploy five clusters with varying cluster size from 10 to 100 in a region of 1300 × 1300 m for measuring energy consumption, computational and storage overheads. The polynomial calculation algorithm is also implemented in Visual Studio 2013 using *C#*, as a programming language. PERL scripts are written to extract the resulting values from the trace file and plot the graph.

### 6.1. Computation Overheads

**Figure 10:**
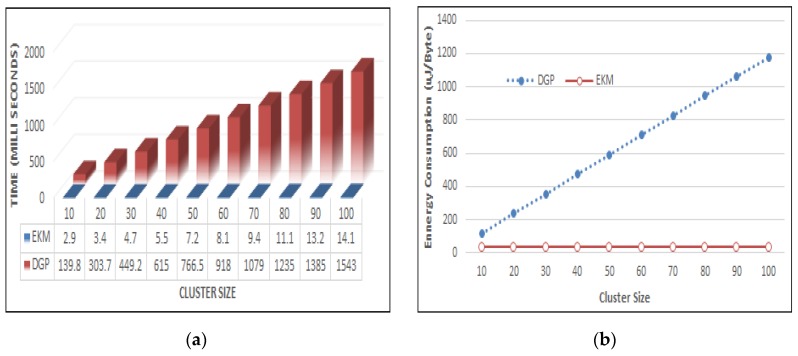
Polynomial computational cost in (**a**) and energy consumptions in (**b**).

We have considered some basic operations v during long multiplication of two numbers *x*, *y.* Computation cost is $\left(2\;\times \;v\right)\times v\;=\;2\;\times v^{2}$ by taking the same count of digits *n* for both numbers. Results of short multiplications are aligned under the respective numbers of *β*. To simplify, we put zeros in the empty positions. Figure 10a shows that a multiplication operation in DGP [18] takes 449.2 significant process shares and *EKM* consumes only 4.7 operation shares. The computational complexity to perform *n-XOR* operations is *O*(*n*) in the *EKM.* The computational complexity of adding two binary numbers is *O*(*n*) whereas that of multiplying two numbers is *O*(*n*^2^). Doubling the number of bits in values *x*, *y*, results in a quadruple value as (2*n*)^2^ = 4*n*^2^.

To compute the hash value of a message using *SHA-1*, a sensor node consumed 5.9 μJ/Byte. For a 16-bit ID, it requires 11.8 μJ. For *n* = 20 member nodes 11.8 × 20 = 236 μJ energy is required just to calculate the hash of IDs. It is directly proportional to cluster size for DGP [18] as illustrated in Figure 10b. In the proposed EKM, only three hash values are randomly selected to calculate the actual key and hence it is independent of the cluster size. This greatly reduces the energy consumption without reducing the security strength.

### 6.2. Latency Time

**Figure 11:**
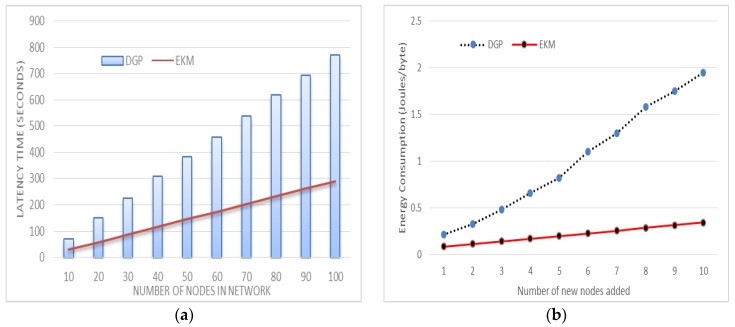
Latency comparisons in (**a**) communication energy in (**b**).

Latency is the time consumed for a data packet to move from one cluster head in the sensor network to the *L*-sensors. Our result in Figure 11a indicates that the latency time is reduced considerably when it is compared with DGP [18]. EKM computed the cluster, key efficiency and reduced the delay on cluster head latency measured by finding the initiating protocol for session key and end time. In the proposed scenario the group head and member nodes participate during session key generation and symmetric parameters consuming less time in establishing a session key for group members. Figure 11a,b show the time delay with respect to group size and the proposed scheme shows less latency which is efficient.

### 6.3. Communication Overhead

Communication cost is measured by taking the ratio of transmitting bytes to received bytes. EKM significantly reduces the number of messages transmitted during new node addition as compared to DGP [18], as depicted in Figure 11b. We have set the transmission cost *T_c_* = 59.2 μJ to transmit and the message receiving cost *R_c_* = 28.6 μJ per byte. The total energy consumed during transmission is $m\times \left(T_{c}\;+\;R_{c}\right)$ where *m* is the number of messages. The lifetime of a sensor network is inversely proportional to its energy consumption. The proposed plan is a very computationally lightweight procedure outlined particularly for the client validation of WSNs in the IoT environment. The method guarantees high security while utilizing just straightforward hash and *XOR* calculations, along these lines, it requires more stockpiling than other related ideas. Since the novelty of the proposed plan is the “sensor-hub first-contact-approach”, together with its high security, the sensor hubs and the *CH* need to convey some additional capacity weight. In this subsection, we display the capacity utilization of our proposed plan and compare it with related ideas.

EKM refreshes keys during node migration, key freshness and node joining more than other similar ones, but it is still below the existing schemes concerning performance and efficiency. The analysis completed by assessing every scheme in detail and calculating the whole length of every variable the *CH* or the sensor node need to save into its memory, while efficiently executing one registration and authentication stage. Before the review, with the end goal of a targeted investigation, we firstly determined the length of each type of a variable, which could be used in the schemes, regardless of the lengths some authors proposed.

**Figure 12:**
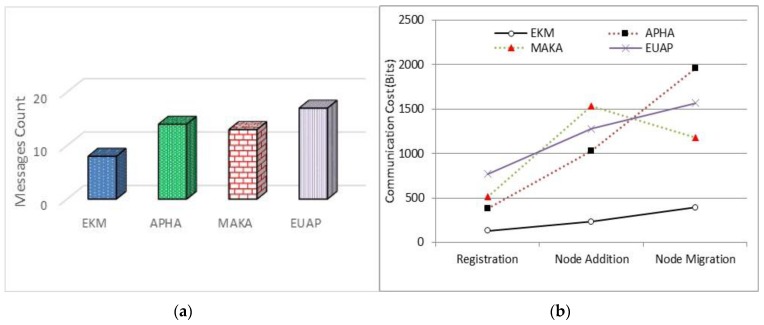
Performance analysis on communication cost where (**a**) represents the message count and (**b**) represents the size of data exchanged.

We have compared our proposed EKM scheme for efficiency of communication and number of of data exchanges in contrast to authentication protocol for healthcare applications (APHA) [44], mutual authentication and key agreement (MAKA) scheme [45] and enhanced user authentication protocol (EUAP) [46] that are recently proposed in IoT scenarios. We used some messages exchanged after deployment of the network and the results show that the proposed protocol is efficient for all possible scenarios. The communication and data used between all participating entities for secure node registration, migration and addition has been considered in Figure 12a,b.

### 6.4. Storage Overhead

**Figure 13:**
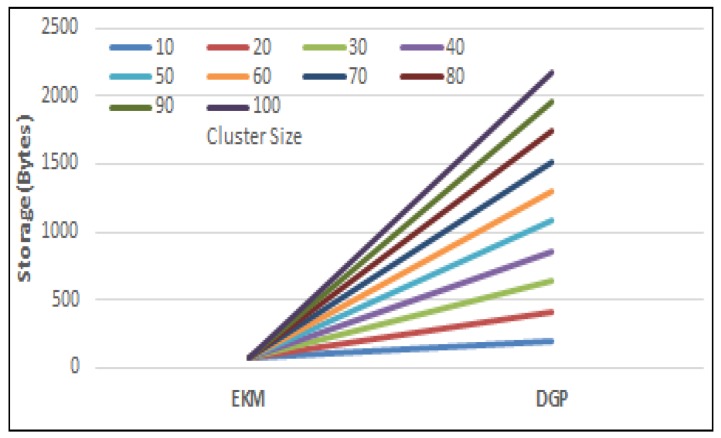
Storage overhead comparison.

The amount of memory required to store security parameters and key space is considered the storage cost. The total number of sensor nodes in a network are nT =nH + nL where *nH* and *nL* represent the total high power nodes and member nodes, respectively. DGP [18] requires multiplication factors F=ID×nL to calculate a polynomial. It stores $\left({\mathrm{ID}}_{B}\times \left(\gamma -1\right)\right)+\left(h{\left(\mathrm{ID}\right)}_{B}\times \left(\gamma -1\right)\right)$. bytes where ${\mathrm{ID}}_{B}$ represents the size of ID in bytes, γ is cluster size, and $h{\left(\mathrm{ID}\right)}_{B}$. is the size of the hash values in bytes. For γ=30, it requires 30 factors to be multiplied for calculating just a one-time polynomial. Moreover, we have considered a value of ${\mathrm{ID}}_{B}=16-$bits and $h{\left(\mathrm{ID}\right)}_{B}=160\;$bits for a secure hash function. It could be much expensive as the cluster size increases. In proposing *EKM*, the suggested value of *F =* 3 and requires 87% less factors as compared to DGP [18] with a cluster size of 30-nodes as illustrated in Figure 13.

### 6.5. Energy Consumption

**Figure 14:**
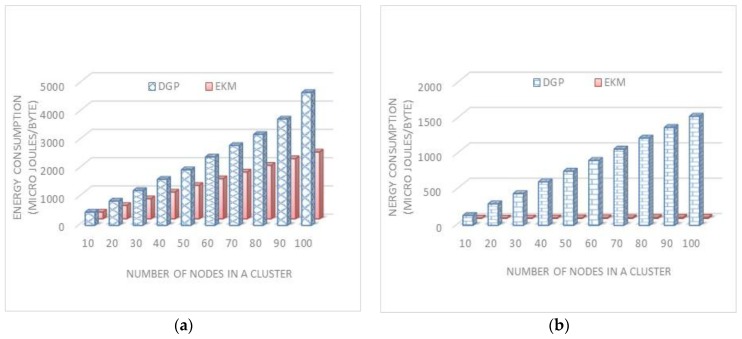
Energy for polynomial generation is presented in (**a**) and key generation energy in (**b**).

To compute the hash value of a message using *SHA-1*, a sensor node consumed 5.9 μJ/Byte. For a 16-bit ID, it requires 11.8 μJ. For *n* = 20 member nodes 11.8 × 20 = 236 μJ energy is required just to calculate the hash of IDs. It is directly proportional to cluster size for DGP [18] as illustrated in Figure 14b. In the proposed EKM, only three hash values are randomly selected to calculate the actual key and hence it is independent of the cluster size. This greatly reduces the energy consumption without reducing the security strength.

The amount of energy required to compute a polynomial by the cluster is linear and existing schemes have a multiple of *n*, where *n* is the number of nodes in a cluster. In the existing technique, cluster head calculates the polynomial after receiving all the ids of the *L*-sensors in its communication range. To compute the secure hash of a sensor node consumes 5.9 μJ/Byte [6]. To compute the hash of *n L*-sensors, the sensor node consumes the bulk of energy. We enhanced the existing technique for a more comprehensive decision about energy consumption for generating polynomials at cluster heads. To compute on a hash of an *L*-sensor id uses 5.9 μJ/Byte, so when we generated polynomials through the existing technique supposing the number of *L*-sensors is n then the consumption of energy is *n* × 5.9 which means it is directly proportional to the increment of *L*-sensors. The simulation of the proposed technique saves four-time more energy than existing techniques. A hash has 128-bit data to be processed which mean that 16-byte × 5.9 μJ is consumed for one way hashing.

These results show that the generation of polynomials through multiplication is more energy consuming than the *XOR*. The number of sensor nodes varies from 10 to 100. The simulation results are shown in Figure 14a,b. We can find that the DGP [18] scheme consumes much more energy as compared to our proposed EKM scheme. This is due to the extensive energy consumption during execution of point multiplication, pairing, and mapping algorithms. Each node needs to run these algorithms many times according to the number of neighbors around the computing node.

## 7. Conclusions

The main objective of the proposed research was to enhance and optimize the polynomial generation during node joining in WSN clusters. In existing schemes, the concept of sequential multiplication was used to calculate the polynomial, which is expensive. This polynomial is recalculated every time a node joins or leaves the cluster, increasing communication, computation and storage overheads. *EKM* is based on generating polynomials using *XOR* of randomly selected values. It increases the lifetime of sensor networks and reduces communication cost and memory overheads. Multiplication operations require a complexity of *O*(*n*^2^) whereas *XOR* requires *O*(*n*)*.* Moreover, the majority of schemes have opted for a centralized approach, where a central node is fully responsible for secure authentication. The proposed research adopted a hybrid approach which is adequate for WSNs as this shares the tasks of the group controller among group members. During our systematic and simulation results, we have exposed that our proposed scheme requires a constant number of hash values to calculate a polynomial whereas in existing schemes that number increases rapidly with cluster size. Our scheme requires 87% less factors for a cluster size of 30 nodes per cluster. In the future, we plan to implement EKM in the Ubiquitous to Internet of Thing (U2IoT) scenario.

## Figures and Tables

**Table 1:** List of Notations for EKM.

Notation	Description
$P_{h_{j}}$	Polynomial Value
$ID_{Li}$	ID of member Node *Li*
$h\left(ID_{Li}\right)$	Hash of Node ID
*γ, n*	Cluster Size, Network Size
$N_{ID_{Li}}$	Array of Node IDs from *i* = 1 to *n*
*R*	Number of Random Hash Values
*CH, BS*	Cluster Head, Base Station
$K_{C–B}$	Key between *CH* and *BS*
$K_{M}$	Master Key
*K_CL_*	Key between *CH* and *Li*
$MAC\left(L_{i}\right)$	Message Authentication Code of *Li*
$LIST_{L_{i}}$	List of ordinary sensors
$A_{h\left(ID\right)}$	Array of Hash values
*P_Li_*	Polynomial for *L_i_*
*M*_1_*–M*_4_	Message 1 to 4
*C*_1_*–C*_3_	Cipher texts of *M*_1_ to *M*_3_
$H_{Li}$	Hash calculated at *L_i_*
$P_{Li},\;P_{CHJ},\;P_{BS}$	Concatenated string at *L_i_*, *CH_j_* and *BS* for transmission

**Table 2:** Local set for EKM.

**1. Sender (Li)**	
POSS(L_i_) = {ID_Li_, K_Li − CHj_} BEL(L_i_) = {#(ID_Li_), #(K_Li − CHj)_ } BL(L_i_) = Hash(h(.); ID_Li_) → H_Li_ Concat(ID_Li_, *nonce_Li_*, *Join-Req*, MAC_ADRS_Li_, H_Li_) → P_Li_ Encrypt({P_Li_}, K_Li − CHj_ ) → C_1_ Send(CH_j_, {ID_Li_, C_1_}) → M_1_ Update(*nonce_Li_*, H_Li_) Receive(L_i_, {ID_CHj_, M_4_}) Split({ID_CHj_, M_4_}) If message does not equal “Join_Success” then abort **2. Cluster Head (CH)** POSS(CH) = {ID_CHj_, K_Li - CHi_, K, K_BS-CHj_, K_M_} BEL(CH) = {#(ID_CHj_), #(K_Li - CHi_), _#_(K_BS-CHj_), #(K_M_)} BL(CH) = Receive(CH_j_, {ID_Li_, C_1_}) Split({ID_Li_, C_1_}) Decrypt({C_1_}, K_CHj-Li_) to get [ID_Li_, *nounce_Li_*, *Join-Req*, MAC_ADRS_Li_, H_Li_] Concat(ID_CHj_, ID_Li_, *nonce_CHj_*, *Ver-Req*, MAC_ADRS_Li_)→P_CHj_ Encrypt({P_CHj_, H_Li_}, K_CHj-BS_ ) → C_2_ Send(BS, {ID_CHj_, C_2_}) → M_2_ Update(M_ID_)	Receive(CH_j_, {ID_BS_, C_3_}) Split({ID_BS_, C_3_}) Decrypt({C_3_}, K_CHj-BS_) to get [ID_BS_, ID_Li_, *Join-Code_T_*, MAC(Concat(ID_BS_, ID_Li_)] Verify *Join-Code_T_* for ID_Li_ If false then abort Add ID_Li_ in node_list Concat(ID_CHj_, ‘Join_Success’) → P_CHj_^~^ Send(BS, {P_CHj_^~^}) → M_4_ **3. Base Station (BS)** POSS(BS) = { ID_BS_, K_BS–CHi_, K_BS–CHj_, List_Nodes} BEL(BS) = {#(ID_BS_), #(K_BS–CHi_), #(K_BS–CHj_)} BL(BS) = Receive(BS, {ID_CHj_, C_2_}) Split({ID_CHj_, C_2_}) Decrypt({C_2_}, K_CHj-BS_) to get [ID_CHj_, ID_Li_, *nonce_CHj_*, *Ver-Req*, MAC_ADRS_Li_, H_Li_] Verify Li and check in Block_List If not verified then Abort Concat(ID_BS_, ID_Li_, *Join-Code_T_*) → P_BS_ Encrypt({P_BS_, MAC(Concat(ID_BS_, ID_Li_)}, K_CHj-BS_) → C_3_ Send(CH_j_, {ID_BS_, C_3_}) → M_3_ Update(M_ID_)
